# Do Size and Insecticide Treatment Matter? Evaluation of Different Nets against *Phlebotomus argentipes*, the Vector of Visceral Leishmaniasis in Nepal

**DOI:** 10.1371/journal.pone.0114915

**Published:** 2014-12-10

**Authors:** Murari Lal Das, Mark Rowland, James W. Austin, Elisa De Lazzari, Albert Picado

**Affiliations:** 1 B.P. Koirala Institute of Health Sciences (BPKIHS), Dharan, Nepal; 2 London School of Hygiene and Tropical Medicine (LSHTM), London, United Kingdom; 3 BASF Corporation, Research Triangle Park, North Carolina, United States of America; 4 ISGlobal, Barcelona Ctr. Int. Health Res. (CRESIB), Hospital Clínic - Universitat de Barcelona, Barcelona, Spain; Universidade Federal do Acre (Federal University of Acre), Brazil

## Abstract

In the Indian subcontinent, *Leishmania donovani*, the parasite causing visceral leishmaniasis (VL) is transmitted by the sand fly vector *Phlebotomus argentipes*. Long lasting insecticide treated nets (LN) have been postulated as alternative or complement to Indoor Residual Spraying but there are few field studies evaluating the entomological efficacy of different nets against this vector. We conducted two crossover trials in a VL endemic area in Nepal to compare the barrier effect of (1) LN with different mesh sizes (156 holes/inch^2^ vs 625 holes/inch^2^) and (2) alpha-cypermethrin treated LN and untreated nets having the same mesh size (156 holes/inch^2^). Each crossover trial had two arms consisting of a sequence of two different nets for 8 nights. We used 10 cattle sheds per trial. A cow placed under the net was used as bait. CDC light traps placed *inside* the nets were used to evaluate the number of *P. argentipes* crossing the net barrier. Negative binomial generalized estimating equation (GEE) population-averaged models adjusted by night and sequence were used to estimate the barrier effect of the different nets. The crossover trials conducted in a rural village in Morang district (South-eastern Nepal) demonstrated that reducing the size of the holes in treated nets (625 holes/inch^2^) increased the barrier effect of LN by 77% (95% confidence interval (CI): 56%–88%) compared with treated nets with larger holes (156 holes/inch^2^). Treating nets with alpha-cypermethrin reduced the number of *P. argentipes* captured inside the nets by 77% (95% CI: 27%–93%) compared with untreated nets. The effectiveness and acceptability of finer mesh pyrethroid treated LN should be tested for VL prevention in a randomized controlled trial.

## Introduction

Visceral leishmaniasis (VL), also known as kala-azar, is a neglected infectious disease caused by protozoa of the *Leishmania donovani* complex transmitted by phlebotomine sand flies. VL is characterized by febrile splenomegaly, pancytopenia, and progressive wasting, which can be fatal if left untreated. VL affects an estimated 200 to 400 thousand people and causes 20 to 40 thousands deaths annually, mostly in Asia and East Africa, and is especially prevalent in poor communities [1].

In India, Nepal and Bangladesh, *L. donovani* is transmitted between people by *Phlebotomus argentipes*. The current vector control strategy in the region based on indoor residual spraying (IRS) with DDT or pyrethroids has been criticized for being costly, not easily accepted, and not sustainable. Bed nets treated with insecticides, and especially long-lasting insecticidal nets (LN), have been proposed as an alternative to IRS [2]. While some observational studies have suggested that untreated nets might also be protective against VL [3], insecticide treated nets (ITN) seem to be more protective against *P. argentipes* than untreated nets [4] and treating bed nets in Bangladesh reduced the sand fly density and the risk of VL [5], [6]. However other studies did not find significant differences in indoor *P. argentipes* density when ITN were deployed [7]. More importantly, the only cluster randomized trial in the region to evaluate the impact of LN on clinical outcomes showed that use of LN did not reduce the risk of *L. donovani* infection or clinical VL in endemic villages of India and Nepal [8]. The reasons for the limited effect of LN against VL in India and Nepal were elusive and may be diverse, with some intrinsic to the vector (e.g. biting and host seeking behaviors, insecticide resistance) and others associated with the nets (e.g. quality, correct use). Some intrinsic to the net were evaluated during the cluster randomized trial [9], [10]. Recent entomological findings, including a trial evaluating the effect of two types of LN on indoor *P. argentipes* in India [7], indicate that *L. donovani* vectors are more exophilic and exophagic than previously reported [11]. This could contribute to the limited impact of ITN on *L. donovani* transmission. However the number of field studies evaluating the effect of different types of net or netting material on *P. argentipes* is still limited. A study in Brazil showed that insecticide treated nets prevent the passage of *Lutzomyia longipalpis*, the principal vector of VL in Latin America compared to untreated nets [12]. But there are no equivalent studies for *P. argentipes* in the Indian subcontinent. The aim of this study was to compare the entomological efficacy of (1) LNs with different mesh sizes (156 vs 625 holes/inch2) and (2) insecticide treated and untreated nets against *P. argentipes* under field conditions in Nepal.

## Materials and Methods

### Study design

Human landing studies are often used to evaluate the barrier capacity of nets in vector borne diseases such as malaria. However, since there is no prophylaxis against VL, such methods are inappropriate for assessing the effect of different types of net (particularly untreated nets) against *P. argentipes* in endemic areas. As an alternative and since *P. argentipes* are known to be opportunistic feeders on cattle and other domestic animals, in addition to feeding on humans [13]–[15], we designed a study using cows as “bait”.

**Figure 1:**
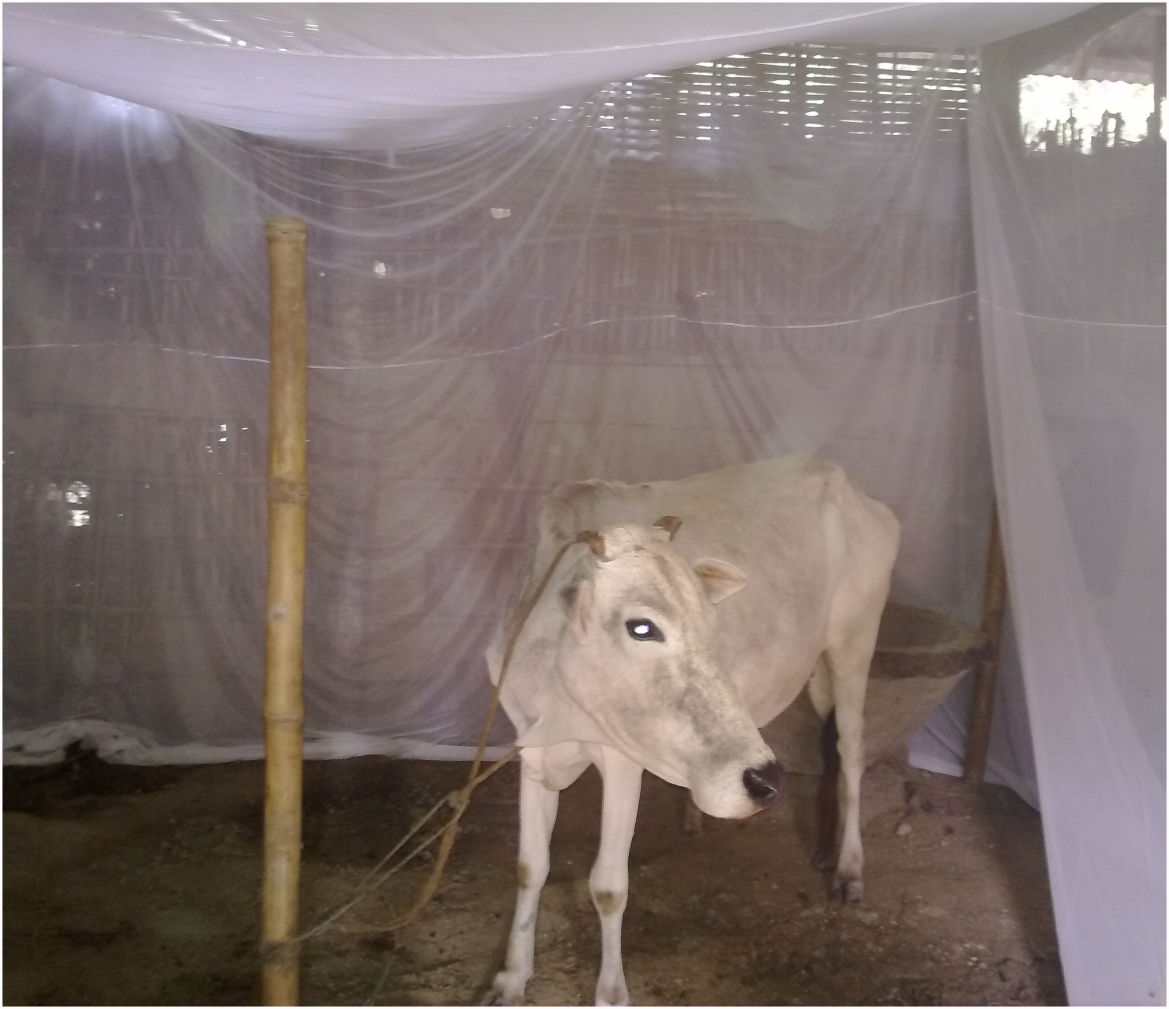
Cow stationed inside one of the nets evaluated in the crossover trial in Nepal.

We conducted two crossover field trials to evaluate the effect of mesh size (Trial 1) and insecticide treatment (Trial 2) against *P. argentipes* in Nepal. Crossover trials use each participant as their own control, reducing confounding variables and increasing the study efficiency compared to parallel group trials. Each crossover trial had two arms consisting of a sequence of two different nets for 8 nights. In each trial, cows were kept under two types of nets (Fig. 1). We evaluated the number of *P. argentipes* inside the nets to compare the barrier effect of the different nets. The two trials were conducted from October to November 2013.

The objective of Trial 1 was to evaluate the impact of mesh size in *P. argentipes’* capacity to cross the net barrier. We compared two types of LN with different mesh sizes 156 holes/inch^2^ (25 holes/cm^2^, net A) and 625 holes/inch^2^ (100 holes/cm^2^, net B). The two study arms were an alternating sequence of nets A and B: Arm 1.1: ABABABAB and Arm 1.2: BABABABA.

The objective of Trial 2 was to evaluate the effect of treating nets with insecticide on *P. argentipes’* capacity to cross the net barrier. We compared long lasting insecticide treated nets (net A) and untreated nets (net C) which had same mesh size (156 holes/inch^2^). The two study arms were an alternating sequence of nets A and C: Arm 2.1: ACACACAC and Arm 2.2: CACACACA.

This crossover design removed the sequence effects [16] and, based on previous studies [12], [17], we did not expect carry-over treatment effects of nets between consecutive nights. The nets, provided by BASF SE, were large enough (500×375×225 cm) to ensure there was enough space under the net for one cow. The insecticide treated nets were factory coated with a proprietary binder system and alpha-cypermethrin: 200 mg/m^2^ and 160 mg/m^2^ for nets A (156 mesh) and B (625 mesh) respectively. All LNs were within the insecticide concentration specifications (150 to 250 mg/m^2^). The 156 mesh alpha-cypermethrin coated nets (Interceptor) produced by BASF are commercially available and possess WHO/WHOPES recommendation as long-lasting insecticidal nets [18].

### Sample size

We recruited a total of 20 cattle sheds, 10 in each crossover trial. The number of cattle sheds required in each trial was calculated estimating a mean difference between the two types of nets of 3 *P. argentipes* (standard deviation of 3) a power of 80% and an alpha of 0.05. These figures were estimated from previous entomological studies [4], [12], [17]. Following the normal approach, the approximate sample size for each of the crossover trial resulted in 10 cattle sheds when the non-central t-distribution was considered [19]. The sample size calculations were conducted using the package xsampsi in Stata v. 12 (StataCorp, College Station, TX, USA).

### Selection of study village and cattle sheds

A village in the Terai, an area endemic for VL in Eastern Nepal, was selected based on the following criteria: *P. argentipes* collected in high numbers in recent years, rural village with enough cattle sheds to conduct the study and no VL cases in the village in the past 5 years. The last criterion was applied to reduce the risk of vector control intervention by IRS by Ministry of Health conducted during the study.

The selection of cattle sheds and cows for the study was done in two steps. First 42 cattle sheds were selected in the study village. Those cattle sheds had to be large enough to deploy the nets. A Baseline survey was conducted in October 2013 placing a CDC light trap in each cattle shed for 1 night. Then, the 20 cattle sheds with the highest number of *P. argentipes* were enrolled into the study.

For each trial, 10 cattle sheds were randomly allocated to one of the study arms. To avoid the possibility of insecticide contamination, the minimum distance between two cattle sheds in each trial was 15 meters. The randomization was done by drawing lots with the cattle shed identification number from a bag.

### Intervention and entomological evaluation

Trial 1 was conducted from the 27^th^ October to the 7^th^ of November and trial 2 from the 8^th^ to the 15^th^ November 2013. Nets were deployed in each cattle shed at 17∶00 and the cows were placed inside the net at 18∶00. The nets were removed during the day.

The number of *P. argentipes* inside the net was determined by means of a CDC light trap placed *inside* the net from 18∶00 to 06∶00. CDC light traps are considered the most efficient and pragmatic method to evaluate *P. argentipes* control interventions as these are attractive to sand flies which enter houses and avoid the need for human involvement or risk [20], but using the light trap to collect sand flies that penetrate inside the net was a novel approach. The sand flies collected were identified under a binocular microscope to species for *Phlebotomus* using keys [21] by a trained entomologist. Gender and presence of blood were also recorded. The entomologist was blinded to type of net used. The entomological outcomes were double entered in an Epi Info database (CDC, GA, USA).

Bioassays were conducted to evaluate the mortality in sand flies exposed to the alpha-cypermethrin treated nets used in the trials (net A = 156 mesh with 200 mg/m^2^ and net B = 625 mesh with 160 mg/m^2^) and a laboratory sample of a 625 mesh LN with 200 mg/m^2^. The standard WHO cone method [22] was used exposing *P. argentipes* to the different LN. Briefly, 10 female sand flies were introduced in 5 cones and exposed for 3 minutes for each LN. Two bioassays were conducted per LN and untreated nets were used as controls. Abbott’s formula was used to calculate the average mortality 24 hours post exposure.

### Statistical analyses

The number of females and total *P. argentipes* captured inside each type of net were compared. The expected count of *P. argentipes* was modelled using negative binomial generalized estimating equation (GEE) population-averaged model. Estimates for net type used (A vs B, and A vs C) were adjusted by night and sequence. The exponential of the estimated coefficient (exp(β)) of net type represents the ratio of the average count of sand flies per night collected under B net over A net, and under A over C. It was interpreted as incidence rate ratio (IRR) and presented as efficacy in percent (1-exp(β))*100. Estimated marginal means were also presented along their standard errors (SE). The confidence level was set at 95% and all tests were two-tailed. We present the expected mean count and standard error (SE) for each net type. We used Stata v13 (StataCorp, College Station, TX, USA) to conduct the statistical analyses.

### Ethical considerations

The study protocol was approved by the Institutional Review Boards and the animal experimentation ethics committee at the University of Barcelona in Spain, B.P. Koirala Institute of Health Sciences in Nepal and Nepal Health Research Council. Informed consent was obtained from cattle shed owners before including their cows and cattle sheds in the study.

## Results

The study was conducted in Bhathigachha, a rural village in Morang district in South-eastern Nepal. Cattle sheds were selected among the 174 houses. The number of *P. argentipes* captured in the 42 cattle sheds initially surveyed for one night in the study village ranged from 0 to 84. At baseline, the cattle sheds selected for trial 1 and trial 2 (10 sheds per trial) had a median of 36 and 45.5 *P. argentipes* collected respectively.

The number of females and total *P. argentipes* captured per night of collection and type of net for trials 1 and 2 is summarized in Table 1. Fewer *P. argentipes* were captured inside the 625 mesh nets (n = 14 *P. argentipes* in net B) compared to 156 mesh nets (n = 61 in net A) in trial 1. Similarly, in trial 2 fewer *P. argentipes* were collected inside the treated nets (n = 27 in net A) than inside untreated nets (n = 77 in net C). Analogous results were observed when only female *P. argentipes* were considered.

**Table 1:** Number of females and total *Phlebotomus argentipes* per capture night and type of net in Trial 1: insecticide treated nets with 156 holes/inch^2^ (A) vs 625 holes/inch^2^ (B) and Trial 2: Insecticide treated nets (A) vs Untreated net (C).

	TRIAL 1				TRIAL 2			
Night of collection	Net	Net sequence	Female *P. argentipes*	Total *P. argentipes*	Net	Net sequence	Female *P. argentipes*	Total *P. argentipes*
1	A	AB	1	17	A	AC	2	3
2	A	BA	7	14	A	CA	4	6
3	A	AB	0	2	A	AC	0	3
4	A	BA	2	3	A	CA	0	2
5	A	AB	0	1	A	AC	0	4
6	A	BA	1	4	A	CA	0	3
7	A	AB	3	6	A	AC	0	6
8	A	BA	2	14	A	CA	0	0
**Total**	**A**		**16**	**61**	**A**		**6**	**27**
1	B	BA	2	5	C	CA	8	26
2	B	AB	1	1	C	AC	5	15
3	B	BA	0	1	C	CA	7	16
4	B	AB	0	1	C	AC	1	1
5	B	BA	1	3	C	CA	0	3
6	B	AB	0	0	C	AC	2	6
7	B	BA	0	1	C	CA	0	3
8	B	AB	0	2	C	AC	2	7
**Total**	**B**		**4**	**14**	**C**		**25**	**77**

The estimated efficacy of net B over net A and net A over C adjusted for net sequence and night are summarized in Table 2. In Trial 1 we observed that the use of 625 mesh size nets (B) reduced by 77% and 78% the number of female *P. argentipes* and total *P. argentipes* captured inside the nets respectively compared to 156 mesh size nets (A). Both nets (A and B) were treated with insecticide. The results of Trial 2 show that using α-cypermethrin treated nets (A) reduced by 77% and 61% the number of female and total *P. argentipes* collected inside the net respectively compared to untreated nets (C). Both nets (A and C) had the same mesh size (156 holes/inch^2^). In both trials the efficacy was statistically significant.

**Table 2:** Results of the negative binomial generalized estimating equation (GEE) population-averaged models estimating the effect of net type on the number *Phlebotomus argentipes* captured inside the nets by CDC light traps, using cattle as bait.

	Female *P. argentipes*			Total *P. argentipes*		
	Adjusted Percent Reduction (95% CI)	p-value	Expected Mean (SE)	Adjusted Percent Reduction (95% CI)	p-value	Expected Mean (SE)
**Trial 1**						
Insecticide treated nets with 156 holes/inch2 (A)	Reference		0.4 (0.20)	Reference		1.55 (0.58)
Insecticide treated nets with 625 holes/inch2 (B)	77% (56%–88%)	<0.0001	0.1 (0.00)	78% (59%–88%)	<0.0001	0.34 (0.16)
**Trial 2**						
Untreated nets with 156 holes/inch2 (C)	Reference		0.65 (0.23)	Reference		1.76 (0.50)
Insecticide treated nets with 156 holes/inch2 (A)	77% (27%–93%)	0.0132	0.15 (0.07)	61% (36%–76%)	0.0001	0.69 (0.16)

Estimates for net type used in each trial (Trial 1: A vs B and Trial 2: A vs C) were adjusted by night and sequence. The effect of nets is presented as efficacy in percent reduction and its 95% confidence interval (CI) for both female and total *P. argentipes*. The marginal estimates: mean count and Standard Error (SE) by net used are also presented.

So the conclusions of the two trials are that (1) 625 mesh size nets perform better than 156 mesh size nets and (2) treated nets perform better than untreated nets against *P. argentipes* under field conditions.

The mean *P. argentipes* mortality per LN was 98% and 94% for the two treated: net A with 200 mg/m^2^ and net B with 160 mg/m^2^ respectively. The laboratory sample of a 625 mesh net with 200 mg/m^2^ of alpha-cypermethrin had an average mortality of 97%. The average control mortality was less than 5%.

## Discussion

The two trials conducted in rural Nepal demonstrate that treating nets with alpha-cypermethrin and reducing the mesh size both significantly increase the barrier effect of the nets against *P. argentipes*. To our knowledge, this is the first study designed to compare the entomological efficacy of different types of net against *L. donovani* vector under field conditions in the Indian subcontinent. The results of this study provide pertinent information to disease control managers, researchers and people living in VL endemic areas in the region considering the use of netting materials (e.g. bed nets, curtains, window screens) against *P. argentipes*. Small mesh size (625 holes/inch^2^), insecticide treated (alpha-cypermethrin) nets provide a better barrier against *P. argentipes* than untreated nets with wider mesh.

Treating standard mesh nets (156 holes/inch^2^) with insecticide reduced the number of *P. argentipes* captured inside the nets by 61%, and by 77% when only females were considered (Trial 2). This finding is supported by a previous field study in India and Nepal where *P. argentipes*’ blood feeding rate was reduced by 85% in sand flies captured indoors when LN (deltamethrin) where deployed compared to the use of untreated nets [4]. But it contrasts with the results of a trial in India where the use of two types of LN did not reduce the number of female *P. argentipes* captured in houses relative to untreated nets [7]. The effect of insecticide treatment of nets has been evaluated against other sand fly vectors. In Brazil, deltamethrin treatment reduced the landing rate of *Lu. Longipalpis* by 39% compared to untreated nets [12]. In Italy and Burkina Faso, the use of permethrin-impregnated curtains in stables and experimental huts reduced the number of sand flies entering them [23], [24]. In Kenya, three types of insecticide treated nets (Olyset LN, PermaNet LN, and K–O Tab treated net) were compared in experimental huts using goats as bait [25] and all three prevented the entry of *P. duboscqi*, the vector of *L. major* in Kenya, relative to untreated nets [25].

Although insecticide treatment can increase the barrier effect of nets, most authorities also recommend evaluating the impact of nets with finer mesh against sand flies [23], [25]. Such studies are, however, scarce. In India, Dinesh *et al* compared nets with different mesh sizes: 156 holes/inch^2^ (PermaNet) and 75 holes/inch^2^ (Olyset Net) but demonstrated no difference in the number of *P. argentipes* captured in houses [7]. Kasili *et al* also compared PermaNet and Olyset Net in Kenya, and in both laboratory and semi-field experiments saw 156 and 75 mesh size preventing sand flies from entering the nets in equal proportions [25]. However, PermaNet and Olyset LN utilize pyrethroids of differing repellency which could confound the effect of mesh size. Our study in Nepal separates the insecticide from mesh effects and shows that using a finer mesh (625 holes/inch^2^) increases significantly the barrier effect of the nets (Trial 1) while the addition of pyrethroid increases the barrier effect (Trial 2). To our knowledge, we are the first team evaluating the entomological efficacy of a 625 mesh size treated net against *Leishmania* vectors under field conditions.

The differences observed between 625 and 156 mesh treated nets are not related to potential differences in alpha-cypermethrin application rate between nets of differing mesh size as shown by the bioassays results.

The use of cows as bait and cattle sheds as the unit of study helped overcoming ethical and methodological issues. We used CDC light traps inside the nets, which are an efficient method for monitoring *P. argentipes* populations [20], to evaluate the entomological efficacy of different nets. The number of sand flies landing on the cows during the night was also evaluated. However, due to the low number of *P. argentipes* captured, the “cow landing rates” were not analyzed independently. The joint analysis of CDC light trap captures and sand flies alighting on cows did not modify the results of the study (see S1 and S2 Tables in S1 File). We have proven that this type of method could be used as a cost-effective means of early field evaluation of vector control tools against *L. donovani* vector in the Indian subcontinent. Selecting cattle sheds that were in use in the village increased the power of the study as high numbers of *P. argentipes* were to be found in them. However their use may have precluded an estimate of the absolute barrier effect of the different types of net since cattle sheds also constitute one of the probable breeding and resting sites for *P. argentipes* [26] and some of the sand flies captured inside the nets may have newly emerged from the substrate beneath the net. However this is unlikely to be a major source of error and would not invalidate the main findings which clearly demonstrated differential efficacy of different type of net against *P. argentipes* in a VL endemic area.

This study shows that treating nets with insecticide and reducing the size of holes improve their barrier effect against *P. argentipes*. Based on these results, the 625 mesh LN would be the preferred option as they perform better than the 156 mesh LN. The latter reduced significantly the risk of contact with *P. argentipes* compared to untreated nets. But, as shown in a recent cluster randomized trial in India and Nepal, the mass distribution of 156 mesh LN did not reduce the risk of VL [8]. The acceptability of 625 mesh LN is presently unclear; bed nets with a smaller mesh size, though more protective, may not be preferred by people living in VL endemic villages if reduced air ventilation is a constraint to use. The effectiveness of finer mesh LN (625 holes/inch^2^) on VL prevention will depend, among other factors, upon the interaction between the perception of increased efficacy and the acceptability to the local population [10] and this trade-off can only be demonstrated by a randomized controlled trial [27].

## Supporting Information

S1 File Evaluation of different nets against *Phlebotomus argentipes* using CDC light trap captures and sand flies alighting on cows as outcome. **S1 Table,** Number of females and total *Phlebotomus argentipes* captured by CDC light trap and mouth aspiration per night and type of net in Trial 1: insecticide treated nets with 156 holes/inch2 (A) vs 625 holes/inch2 (B) and Trial 2: Insecticide treated nets (A) vs Untreated net (C). **S2 Table,** Results of the negative binomial generalized estimating equation (GEE) population-averaged models estimating the effect of net type on the number *Phlebotomus argentipes* captured inside the nets by CDC light traps and mouth aspiration, using cattle as bait. Estimates for net type used in each trial (Trial 1: A vs B and Trial 2: A vs C) were adjusted by night and sequence. The effect of nets is presented as efficacy in percent reduction and its 95% confidence interval (CI) for both female and total *P. argentipes*.
(DOCX)
