# Supplementary material for: Do Size and Insecticide Treatment Matter? Evaluation of Different Nets against Phlebotomus argentipes, the Vector of Visceral Leishmaniasis in Nepal
Source: PLoS One. 2014 Dec 10;9(12):e114915. doi: 10.1371/journal.pone.0114915 (PMC4262434; doi:10.1371/journal.pone.0114915)
Supplement: S1 File — Evaluation of different nets against Phlebotomus argentipes using CDC light trap captures and sand flies alighting on cows as outcome. S1 Table, Number of females and total Phlebotomus argentipes captured by CDC light trap and mouth aspiration per night and type of net in Trial 1: insecticide treated nets with 156 holes/inch2 (A) vs 625 holes/inch2 (B) and Trial 2: Insecticide treated nets (A) vs Untreated net (C). S2 Table, Results of the negative binomial generalized estimating equation (GEE) population-averaged models estimating the effect of net type on the number Phlebotomus argentipes captured inside the nets by CDC light traps and mouth aspiration, using cattle as bait. Estimates for net type used in each trial (Trial 1: A vs B and Trial 2: A vs C) were adjusted by night and sequence. The effect of nets is presented as efficacy in percent reduction and its 95% confidence interval (CI) for both female and total P. argentipes. (DOCX) [file pone.0114915.s001.docx]

**Supporting Information**

Evaluation of different nets against *Phlebotomus argentipes* using CDC light trap captures and sand flies alighting on cows as outcome

**Materials and Methods**

Sand flies landing on the study cows during the night were captured by mouth aspiration. Cows were monitored for 10 minutes per hour from 19:00 to 03:00 by experienced field workers. The field workers rotated among cows each night to reduce the risk of bias. Sand flies captured by mouth aspiration were identified as described in the main article. The *P. argentipes* captured by CDC light trap and mouth aspiration per cow and night were added and analysed as described in the main paper.

**Results**

Few *P. argentipes* were captured by mouth aspiration in both trials: 5 in Trial 1 (3 in net A and 2 in net B), and 4 in Trial 2 (0 in net A and 4 in net C). All *P. argentipes* captured by mouth aspiration were females. Table S1 summarises the number of females and total *P. argentipes* captured per CDC light trap and mouth aspiration per night and type of net.

**Table S1:** Number of females and total *Phlebotomus argentipes* captured by CDC light trap and mouth aspiration per night and type of net in Trial 1: insecticide treated nets with 156 holes/inch^2^ (A) vs 625 holes/inch^2^ (B) and Trial 2: Insecticide treated nets (A) vs Untreated net (C).

|  | TRIAL 1 | | | |  | TRIAL 2 | | | |
| --- | --- | --- | --- | --- | --- | --- | --- | --- | --- |
| Night of collection | Net | Net sequence | Female *P. argentipes* | Total *P. argentipes* |  | Net | Net sequence | Female *P. argentipes* | Total *P. argentipes* |
| 1 | A | AB | 1 | 17 |  | A | AC | 2 | 3 |
| 2 | A | BA | 8 | 15 |  | A | CA | 4 | 6 |
| 3 | A | AB | 0 | 2 |  | A | AC | 0 | 3 |
| 4 | A | BA | 2 | 3 |  | A | CA | 0 | 2 |
| 5 | A | AB | 0 | 1 |  | A | AC | 0 | 4 |
| 6 | A | BA | 1 | 4 |  | A | CA | 0 | 3 |
| 7 | A | AB | 3 | 6 |  | A | AC | 0 | 6 |
| 8 | A | BA | 4 | 16 |  | A | CA | 0 | 0 |
| **Total** | **A** |  | **19** | **64** |  | **A** |  | **6** | **27** |
| 1 | B | BA | 3 | 6 |  | C | CA | 10 | 28 |
| 2 | B | AB | 1 | 1 |  | C | AC | 6 | 16 |
| 3 | B | BA | 1 | 2 |  | C | CA | 8 | 17 |
| 4 | B | AB | 0 | 1 |  | C | AC | 1 | 1 |
| 5 | B | BA | 1 | 3 |  | C | CA | 0 | 3 |
| 6 | B | AB | 0 | 0 |  | C | AC | 2 | 6 |
| 7 | B | BA | 0 | 1 |  | C | CA | 0 | 3 |
| 8 | B | AB | 0 | 2 |  | C | AC | 2 | 7 |
| **Total** | **B** |  | **6** | **16** |  | **C** |  | **29** | **81** |

When the CDC light traps and mouth aspiration captures were analysed together, the results of the GEE population-averaged regression models (Table S2) were similar to those reported in the main paper where only CDC light trap captures were considered. In Trial 1 we observed that using 625 mesh size nets (B) reduced by 72% and 80% the number of female *P. argentipes* and total *P. argentipes* captured inside the nets respectively compared to 156 mesh size nets (A). The results of Trial 2 show that using α-cypermethrin treated nets (A) reduced by 77% and 62% the number of female and total *P. argentipes* collected inside the net respectively compared to untreated nets (C).

**Table S2:** Results of the negative binomial generalized estimating equation (GEE) population-averaged models estimating the effect of net type on the number *Phlebotomus argentipes* captured inside the nets by CDC light traps and mouth aspiration, using cattle as bait. Estimates for net type used in each trial (Trial 1: A vs B and Trial 2: A vs C) were adjusted by night and sequence. The effect of nets is presented as efficacy in percent reduction and its 95% confidence interval (CI) for both female and total *P. argentipes*.

|  | Female *P. argentipes* | |  | Total *P. argentipes* | |
| --- | --- | --- | --- | --- | --- |
|  | Adjusted Percent Reduction (95%CI) | p-value |  | Adjusted Percent Reduction (95%CI) | p-value |
| **Trial 1** |  |  |  |  |  |
| Insecticide treated nets with 156 holes/inch^2^ (A) | Reference |  |  | Reference |  |
| Insecticide treated nets with 625 holes/inch^2^  (B) | 72% (54% - 83%) | < 0.0001 |  | 77% (57% - 87%) | < 0.0001 |
|  |  |  |  |  |  |
| **Trial 2** |  |  |  |  |  |
| Untreated nets with 156 holes/inch^2^ (C) | Reference |  |  | Reference |  |
| Insecticide treated nets with 156 holes/inch^2^ (A) | 80% (37% - 94%) | 0.0062 |  | 62% (36% - 78%) | 0.0003 |
